# Crystal structure of 3-[(*E*)-2-(4-phenyl-1,3-thia­zol-2-yl)hydrazin-1-yl­idene]indolin-2-one

**DOI:** 10.1107/S1600536814022715

**Published:** 2014-10-24

**Authors:** Bhimashankar M. Halasangi, Prema S. Badami, Sangamesh A. Patil, G. N. Anil Kumar

**Affiliations:** a Department of Chemistry, Karnatak University, Dharwad, India; b Department of Chemistry, Shri Sharanabasaveshwar College of Science, Gulbarga 585 102, India; c Department of Physics, M S Ramaiah Institute of Technology, Bangalore 560 054, Karnataka, India

**Keywords:** crystal structure, indolinone, hydrazine, 1,3-thia­zole, hydrogen bonding, biological activity

## Abstract

In the title mol­ecule, C_17_H_12_N_4_OS, the thia­zole ring forms a dihedral angle of 10.8 (2)° with the phenyl ring and an angle of 3.1 (3)° with the indole ring system [which has a maximum deviation of 0.035 (2) Å]. The dihedral angle between the planes of the phenyl ring and the indole ring system is 11.5 (1)°. An intra­molecular N—H⋯O hydrogen bond is observed. In the crystal, pairs of N—H⋯O hydrogen bonds form inversion dimers with an *R* ^2^_2_(8) graph-set motif.

## Related literature   

For the biological activities of substituted thia­zoles, see: Ali *et al.* (2011 ▶); Bharti *et al.* (2010 ▶); Kondratieva *et al.* (2007 ▶). For a related structure, see: Sadık *et al.* (2004 ▶).
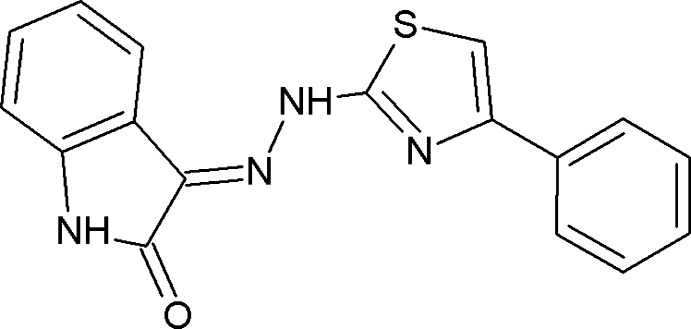


## Experimental   

### Crystal data  

C_17_H_12_N_4_OS
*M* *_r_* = 320.37
Monoclinic, 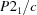

*a* = 17.7108 (8) Å
*b* = 5.1411 (2) Å
*c* = 15.9065 (6) Å
β = 94.706 (3)°
*V* = 1443.45 (10) Å^3^
*Z* = 4
Mo *K*α radiation
μ = 0.23 mm^−1^
*T* = 296 K
0.35 × 0.31 × 0.25 mm

### Data collection  

Bruker SMART CCD area-detector diffractometer
Absorption correction: multi-scan (*SADABS*; Sheldrick, 1996 ▶) *T*_min_ = 0.887, *T*_max_ = 0.934
11530 measured reflections
3142 independent reflections
2124 reflections with *I* > 2σ(*I*)
*R*_int_ = 0.039

### Refinement  

*R*[*F* ^2^ > 2σ(*F* ^2^)] = 0.046
*wR*(*F* ^2^) = 0.109
*S* = 1.09
3142 reflections
208 parameters
H-atom parameters constrained
Δρ_max_ = 0.20 e Å^−3^
Δρ_min_ = −0.25 e Å^−3^

### 

Data collection: *SMART* (Bruker, 1998 ▶); cell refinement: *SAINT* (Bruker, 1998 ▶); data reduction: *SAINT*; program(s) used to solve structure: *SIR92* (Altomare *et al.*, 1994 ▶); program(s) used to refine structure: *SHELXL97* (Sheldrick, 2008 ▶); molecular graphics: *ORTEP-3 for Windows* (Farrugia, 2012 ▶) and *CAMERON* (Watkin *et al.*, 1993 ▶); software used to prepare material for publication: *PARST* (Nardelli, 1995 ▶) and *PLATON* (Spek, 2009 ▶).

## Supplementary Material

Crystal structure: contains datablock(s) I. DOI: 10.1107/S1600536814022715/lh5732sup1.cif

Structure factors: contains datablock(s) I. DOI: 10.1107/S1600536814022715/lh5732Isup2.hkl

Supporting information file. DOI: 10.1107/S1600536814022715/lh5732Isup3.cml

**Fig. 1.**
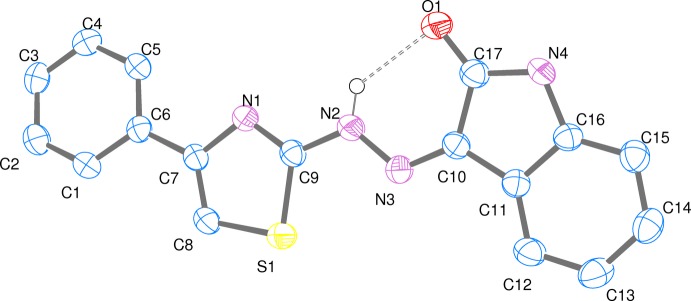
The molecular structure of the title compound with displacement ellipsoids drawn at the 50% probability level. The dashed line indicates an intramolecular N—H···N bond

. DOI: 10.1107/S1600536814022715/lh5732fig1.tif
The mol­ecular structure of the title compound with displacement ellipsoids drawn at the 50% probability level. The dashed line indicates an intra­molecular N—H⋯N bond

**Fig. 2.**
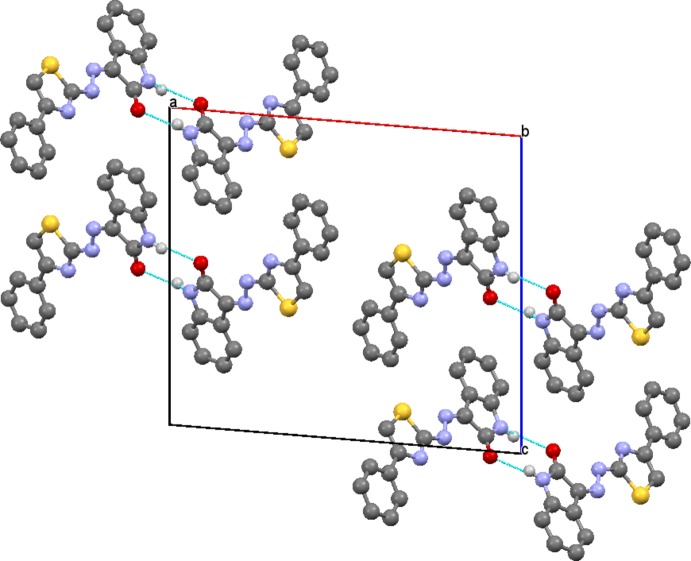
Part of the crystal structure with hydrogen bonds indicated as dotted lines

. DOI: 10.1107/S1600536814022715/lh5732fig2.tif
Part of the crystal structure with hydrogen bonds indicated as dotted lines

CCDC reference: 1029498

Additional supporting information:  crystallographic information; 3D view; checkCIF report

## Figures and Tables

**Table 1:** Hydrogen-bond geometry (, )

*D*H*A*	*D*H	H*A*	*D* *A*	*D*H*A*
N2H2O1	0.86	2.12	2.771(2)	133
N4H4O1^i^	0.86	2.11	2.922(2)	158

Symmetry code: (i) 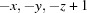.

## supplementary crystallographic information

### S1.1. Synthesis and crystallization

An ethano­lic solution of 1.81g (0.01 M) of 2-hydrazino-4-phenyl­thia­zole was added drop wise to an etanolic solution of 1.47g (0.01 M) of isatin with constant stirring. After the complete addition, the reaction mixture was stirred further for 8-9 hrs until the solid separated out from the reaction mixture. The separated solid was filtered and washed with cold alcohol, dried and recrystallized from DMF (Yield: 95 %. MP: 443-446K). Block-shaped colourless crystals were obtained by slow evaporation of a solution of the title compound at room temperature in DMF:water in the ratio 2:1.

### S1.2. Refinement

H atoms were placed in idealized positions and refined using a riding-model approximation with N—H = 0.86 Å, C—H = 0.93 Å and with U_iso_(H) = 1.2U_eq_(N,C).

### S2. Comment

Isatin derivatives and compounds containing a thia­zole ring are class of organic compounds which have fascinated many synthetic researchers due to their wide range of biological activity ( Ali *et al.*, 2011; Bharti *et al.*, 2010; Kondratieva *et al.*, 2007).

The molecular structure of the title compound is shown in Fig. 1. An intra­molecular N—H···O hydrogen bond is observed. The thia­zole ring is essentially planar with a maximum deviation of 0.005 (2) Å for atom N1. The thia­zole ring (S1/C9/N1/C7/C8) forms dihedral angles of 10.8 (2)° with the phenyl ring (C1–C6) and 3.1 (3)° with the indole ring system (C10—C16/N4/C17, with a maximum deviation of 0.035 (2)Å for atom C17). The dihedral angle between the phenyl ring and the indole ring system is 11.5 (1)Å. In the crystal, pairs of N—H···O hydrogen bonds form inversion dimers (Fig. 2). A closely related structure appears in the literature (Sadik, *et al.*, 2004).

### Crystal data

**Table:**

C_17_H_12_N_4_OS	*Z* = 4
*M**_r_* = 320.37	*F*(000) = 664
Monoclinic, *P*2_1_/*c*	*D*_x_ = 1.474 Mg m^−^^3^
Hall symbol: -P 2ybc	Mo *K*α radiation, λ = 0.71073 Å
*a* = 17.7108 (8) Å	µ = 0.23 mm^−^^1^
*b* = 5.1411 (2) Å	*T* = 296 K
*c* = 15.9065 (6) Å	Block, colourless
β = 94.706 (3)°	0.35 × 0.31 × 0.25 mm
*V* = 1443.45 (10) Å^3^	

### Data collection

**Table:**

Bruker SMART CCD area-detector diffractometer	2124 reflections with *I* > 2σ(*I*)
Graphite monochromator	*R*_int_ = 0.039
φ and ω scans	θ_max_ = 27.1°, θ_min_ = 2.3°
Absorption correction: multi-scan (*SADABS*; Sheldrick, 1996)	*h* = −22→22
*T*_min_ = 0.887, *T*_max_ = 0.934	*k* = −6→6
11530 measured reflections	*l* = −20→20
3142 independent reflections	

### Refinement

**Table:**

Refinement on *F*^2^	Primary atom site location: structure-invariant direct methods
Least-squares matrix: full	Secondary atom site location: difference Fourier map
*R*[*F*^2^ > 2σ(*F*^2^)] = 0.046	Hydrogen site location: inferred from neighbouring sites
*wR*(*F*^2^) = 0.109	H-atom parameters constrained
*S* = 1.09	*w* = 1/[σ^2^(*F*_o_^2^) + (0.0451*P*)^2^ + 0.0098*P*] where *P* = (*F*_o_^2^ + 2*F*_c_^2^)/3
3142 reflections	(Δ/σ)_max_ < 0.001
208 parameters	Δρ_max_ = 0.20 e Å^−^^3^
0 restraints	Δρ_min_ = −0.25 e Å^−^^3^

### Special details

**Table:**

Geometry. All e.s.d.'s (except the e.s.d. in the dihedral angle between two l.s. planes) are estimated using the full covariance matrix. The cell e.s.d.'s are taken into account individually in the estimation of e.s.d.'s in distances, angles and torsion angles; correlations between e.s.d.'s in cell parameters are only used when they are defined by crystal symmetry. An approximate (isotropic) treatment of cell e.s.d.'s is used for estimating e.s.d.'s involving l.s. planes.
Refinement. Refinement of *F*^2^ against ALL reflections. The weighted *R*-factor *wR* and goodness of fit *S* are based on *F*^2^, conventional *R*-factors *R* are based on *F*, with *F* set to zero for negative *F*^2^. The threshold expression of *F*^2^ > σ(*F*^2^) is used only for calculating *R*-factors(gt) *etc*. and is not relevant to the choice of reflections for refinement. *R*-factors based on *F*^2^ are statistically about twice as large as those based on *F*, and *R*- factors based on ALL data will be even larger.

### Fractional atomic coordinates and isotropic or equivalent isotropic displacement parameters (Å^2^)

**Table:**

	*x*	*y*	*z*	*U*_iso_*/*U*_eq_	
S1	0.33816 (3)	0.66660 (12)	0.39879 (3)	0.04097 (19)	
O1	0.08908 (8)	0.2212 (3)	0.51706 (8)	0.0481 (4)	
N1	0.28727 (8)	0.7985 (3)	0.54037 (9)	0.0325 (4)	
N2	0.21944 (9)	0.4599 (3)	0.46996 (9)	0.0369 (4)	
H2	0.1873	0.4489	0.5075	0.044*	
N3	0.21516 (9)	0.3013 (3)	0.40283 (9)	0.0341 (4)	
N4	0.05982 (9)	−0.1170 (3)	0.42364 (9)	0.0387 (5)	
H4	0.0225	−0.1842	0.4474	0.046*	
C1	0.43038 (11)	1.3281 (4)	0.58140 (12)	0.0359 (5)	
H1	0.4504	1.335	0.5292	0.043*	
C2	0.45583 (11)	1.5006 (4)	0.64372 (12)	0.0419 (5)	
H2A	0.4931	1.6214	0.6335	0.05*	
C3	0.42631 (12)	1.4945 (4)	0.72089 (12)	0.0410 (5)	
H3	0.4433	1.6113	0.7629	0.049*	
C4	0.37150 (12)	1.3150 (4)	0.73568 (12)	0.0415 (5)	
H4A	0.3512	1.3113	0.7877	0.05*	
C5	0.34649 (11)	1.1406 (4)	0.67384 (12)	0.0376 (5)	
H5	0.3098	1.0188	0.6849	0.045*	
C6	0.37525 (10)	1.1438 (4)	0.59528 (11)	0.0304 (5)	
C7	0.34960 (10)	0.9552 (4)	0.52935 (11)	0.0309 (5)	
C8	0.38282 (11)	0.9102 (4)	0.45680 (11)	0.0367 (5)	
H8	0.4247	1.0007	0.4406	0.044*	
C9	0.27677 (11)	0.6406 (4)	0.47676 (11)	0.0314 (5)	
C10	0.16106 (11)	0.1315 (4)	0.39588 (11)	0.0319 (5)	
C11	0.14763 (10)	−0.0560 (4)	0.32827 (11)	0.0317 (5)	
C12	0.18154 (11)	−0.1039 (4)	0.25430 (12)	0.0409 (5)	
H12	0.222	−0.003	0.2397	0.049*	
C13	0.15406 (12)	−0.3045 (4)	0.20274 (12)	0.0451 (6)	
H13	0.1762	−0.3386	0.1528	0.054*	
C14	0.09393 (12)	−0.4556 (4)	0.22454 (12)	0.0422 (6)	
H14	0.0768	−0.5912	0.1893	0.051*	
C15	0.05887 (11)	−0.4091 (4)	0.29754 (12)	0.0392 (5)	
H15	0.0183	−0.51	0.312	0.047*	
C16	0.08638 (10)	−0.2078 (4)	0.34775 (11)	0.0319 (5)	
C17	0.10069 (11)	0.0896 (4)	0.45404 (12)	0.0362 (5)	

### Atomic displacement parameters (Å^2^)

**Table:**

	*U*^11^	*U*^22^	*U*^33^	*U*^12^	*U*^13^	*U*^23^
S1	0.0424 (3)	0.0466 (4)	0.0351 (3)	−0.0080 (3)	0.0099 (2)	−0.0052 (2)
O1	0.0479 (9)	0.0568 (11)	0.0417 (8)	−0.0142 (8)	0.0156 (7)	−0.0154 (8)
N1	0.0296 (9)	0.0333 (11)	0.0350 (8)	−0.0022 (8)	0.0050 (7)	−0.0018 (8)
N2	0.0329 (10)	0.0424 (12)	0.0363 (9)	−0.0091 (9)	0.0080 (7)	−0.0058 (8)
N3	0.0328 (9)	0.0359 (11)	0.0335 (8)	−0.0026 (9)	0.0026 (7)	−0.0024 (8)
N4	0.0354 (10)	0.0422 (12)	0.0400 (9)	−0.0129 (9)	0.0112 (7)	−0.0035 (8)
C1	0.0343 (11)	0.0375 (14)	0.0362 (10)	−0.0027 (10)	0.0056 (8)	0.0039 (10)
C2	0.0377 (12)	0.0398 (15)	0.0478 (12)	−0.0074 (11)	0.0010 (9)	−0.0001 (11)
C3	0.0436 (13)	0.0342 (14)	0.0442 (12)	−0.0026 (11)	−0.0030 (9)	−0.0071 (10)
C4	0.0454 (13)	0.0432 (15)	0.0366 (11)	−0.0002 (12)	0.0078 (9)	−0.0046 (10)
C5	0.0359 (12)	0.0366 (14)	0.0411 (11)	−0.0069 (11)	0.0085 (9)	−0.0020 (10)
C6	0.0291 (11)	0.0275 (12)	0.0343 (10)	0.0043 (10)	0.0010 (8)	0.0020 (9)
C7	0.0289 (10)	0.0285 (12)	0.0354 (10)	−0.0006 (10)	0.0026 (8)	0.0023 (9)
C8	0.0345 (11)	0.0377 (14)	0.0385 (11)	−0.0086 (10)	0.0071 (9)	−0.0001 (10)
C9	0.0290 (11)	0.0309 (13)	0.0344 (10)	−0.0004 (10)	0.0026 (8)	0.0012 (9)
C10	0.0282 (11)	0.0341 (13)	0.0334 (10)	−0.0010 (10)	0.0025 (8)	0.0010 (9)
C11	0.0275 (10)	0.0331 (13)	0.0342 (10)	0.0012 (10)	0.0016 (8)	−0.0001 (9)
C12	0.0326 (12)	0.0505 (15)	0.0404 (11)	−0.0049 (11)	0.0080 (9)	−0.0022 (11)
C13	0.0370 (12)	0.0588 (17)	0.0398 (11)	0.0042 (12)	0.0047 (9)	−0.0104 (11)
C14	0.0381 (12)	0.0428 (15)	0.0445 (12)	0.0027 (11)	−0.0042 (9)	−0.0094 (11)
C15	0.0343 (12)	0.0387 (14)	0.0442 (11)	−0.0016 (11)	0.0013 (9)	−0.0029 (10)
C16	0.0279 (11)	0.0358 (13)	0.0321 (10)	0.0026 (10)	0.0026 (8)	−0.0003 (9)
C17	0.0322 (11)	0.0398 (14)	0.0368 (11)	−0.0016 (11)	0.0040 (9)	−0.0008 (10)

### Geometric parameters (Å, º)

**Table:**

S1—C8	1.711 (2)	C4—C5	1.377 (3)
S1—C9	1.7203 (19)	C4—H4A	0.93
O1—C17	1.240 (2)	C5—C6	1.388 (2)
N1—C9	1.299 (2)	C5—H5	0.93
N1—C7	1.389 (2)	C6—C7	1.472 (3)
N2—N3	1.341 (2)	C7—C8	1.358 (2)
N2—C9	1.374 (2)	C8—H8	0.93
N2—H2	0.86	C10—C11	1.449 (3)
N3—C10	1.294 (2)	C10—C17	1.486 (3)
N4—C17	1.352 (2)	C11—C12	1.386 (2)
N4—C16	1.411 (2)	C11—C16	1.392 (3)
N4—H4	0.86	C12—C13	1.381 (3)
C1—C2	1.378 (3)	C12—H12	0.93
C1—C6	1.391 (3)	C13—C14	1.385 (3)
C1—H1	0.93	C13—H13	0.93
C2—C3	1.373 (3)	C14—C15	1.382 (3)
C2—H2A	0.93	C14—H14	0.93
C3—C4	1.373 (3)	C15—C16	1.372 (3)
C3—H3	0.93	C15—H15	0.93
			
C8—S1—C9	87.69 (9)	C7—C8—S1	111.71 (15)
C9—N1—C7	109.19 (15)	C7—C8—H8	124.1
N3—N2—C9	117.83 (15)	S1—C8—H8	124.1
N3—N2—H2	121.1	N1—C9—N2	122.80 (17)
C9—N2—H2	121.1	N1—C9—S1	117.03 (15)
C10—N3—N2	118.11 (16)	N2—C9—S1	120.17 (14)
C17—N4—C16	111.08 (16)	N3—C10—C11	125.96 (17)
C17—N4—H4	124.5	N3—C10—C17	127.60 (18)
C16—N4—H4	124.5	C11—C10—C17	106.44 (17)
C2—C1—C6	121.10 (18)	C12—C11—C16	119.27 (18)
C2—C1—H1	119.5	C12—C11—C10	133.79 (19)
C6—C1—H1	119.5	C16—C11—C10	106.93 (16)
C3—C2—C1	120.2 (2)	C13—C12—C11	118.73 (19)
C3—C2—H2A	119.9	C13—C12—H12	120.6
C1—C2—H2A	119.9	C11—C12—H12	120.6
C2—C3—C4	119.63 (19)	C12—C13—C14	120.71 (19)
C2—C3—H3	120.2	C12—C13—H13	119.6
C4—C3—H3	120.2	C14—C13—H13	119.6
C3—C4—C5	120.37 (19)	C15—C14—C13	121.4 (2)
C3—C4—H4A	119.8	C15—C14—H14	119.3
C5—C4—H4A	119.8	C13—C14—H14	119.3
C4—C5—C6	121.01 (19)	C16—C15—C14	117.20 (19)
C4—C5—H5	119.5	C16—C15—H15	121.4
C6—C5—H5	119.5	C14—C15—H15	121.4
C5—C6—C1	117.73 (18)	C15—C16—C11	122.65 (18)
C5—C6—C7	121.26 (18)	C15—C16—N4	128.34 (18)
C1—C6—C7	121.00 (17)	C11—C16—N4	109.01 (17)
C8—C7—N1	114.38 (17)	O1—C17—N4	126.73 (19)
C8—C7—C6	126.00 (18)	O1—C17—C10	126.84 (19)
N1—C7—C6	119.59 (16)	N4—C17—C10	106.42 (17)
			
C9—N2—N3—C10	−179.69 (17)	N2—N3—C10—C17	0.7 (3)
C6—C1—C2—C3	−0.6 (3)	N3—C10—C11—C12	−3.6 (4)
C1—C2—C3—C4	0.2 (3)	C17—C10—C11—C12	176.1 (2)
C2—C3—C4—C5	0.4 (3)	N3—C10—C11—C16	177.11 (18)
C3—C4—C5—C6	−0.7 (3)	C17—C10—C11—C16	−3.2 (2)
C4—C5—C6—C1	0.3 (3)	C16—C11—C12—C13	−1.0 (3)
C4—C5—C6—C7	179.04 (18)	C10—C11—C12—C13	179.7 (2)
C2—C1—C6—C5	0.3 (3)	C11—C12—C13—C14	−0.2 (3)
C2—C1—C6—C7	−178.37 (17)	C12—C13—C14—C15	0.9 (3)
C9—N1—C7—C8	0.9 (2)	C13—C14—C15—C16	−0.3 (3)
C9—N1—C7—C6	−177.30 (17)	C14—C15—C16—C11	−0.9 (3)
C5—C6—C7—C8	−167.50 (19)	C14—C15—C16—N4	178.32 (18)
C1—C6—C7—C8	11.2 (3)	C12—C11—C16—C15	1.6 (3)
C5—C6—C7—N1	10.4 (3)	C10—C11—C16—C15	−178.92 (17)
C1—C6—C7—N1	−170.89 (17)	C12—C11—C16—N4	−177.75 (17)
N1—C7—C8—S1	−0.5 (2)	C10—C11—C16—N4	1.7 (2)
C6—C7—C8—S1	177.49 (15)	C17—N4—C16—C15	−178.68 (18)
C9—S1—C8—C7	0.06 (16)	C17—N4—C16—C11	0.7 (2)
C7—N1—C9—N2	179.57 (17)	C16—N4—C17—O1	176.05 (19)
C7—N1—C9—S1	−0.8 (2)	C16—N4—C17—C10	−2.7 (2)
N3—N2—C9—N1	−178.05 (17)	N3—C10—C17—O1	4.6 (3)
N3—N2—C9—S1	2.4 (2)	C11—C10—C17—O1	−175.10 (19)
C8—S1—C9—N1	0.48 (16)	N3—C10—C17—N4	−176.72 (18)
C8—S1—C9—N2	−179.92 (16)	C11—C10—C17—N4	3.6 (2)
N2—N3—C10—C11	−179.72 (16)		

### Hydrogen-bond geometry (Å, º)

**Table:**

*D*—H···*A*	*D*—H	H···*A*	*D*···*A*	*D*—H···*A*
N2—H2···O1	0.86	2.12	2.771 (2)	133
N4—H4···O1^i^	0.86	2.11	2.922 (2)	158

Symmetry code: (i) −*x*, −*y*, −*z*+1.
