# Supplementary figures and images for: Crystal structure of 3-[(E)-2-(4-phenyl-1,3-thia­zol-2-yl)hydrazin-1-yl­idene]indolin-2-one
Source: Acta Crystallogr Sect E Struct Rep Online. 2014 Oct 24;70(Pt 11):o1177–8. doi: 10.1107/S1600536814022715 (PMC4257251; doi:10.1107/S1600536814022715)

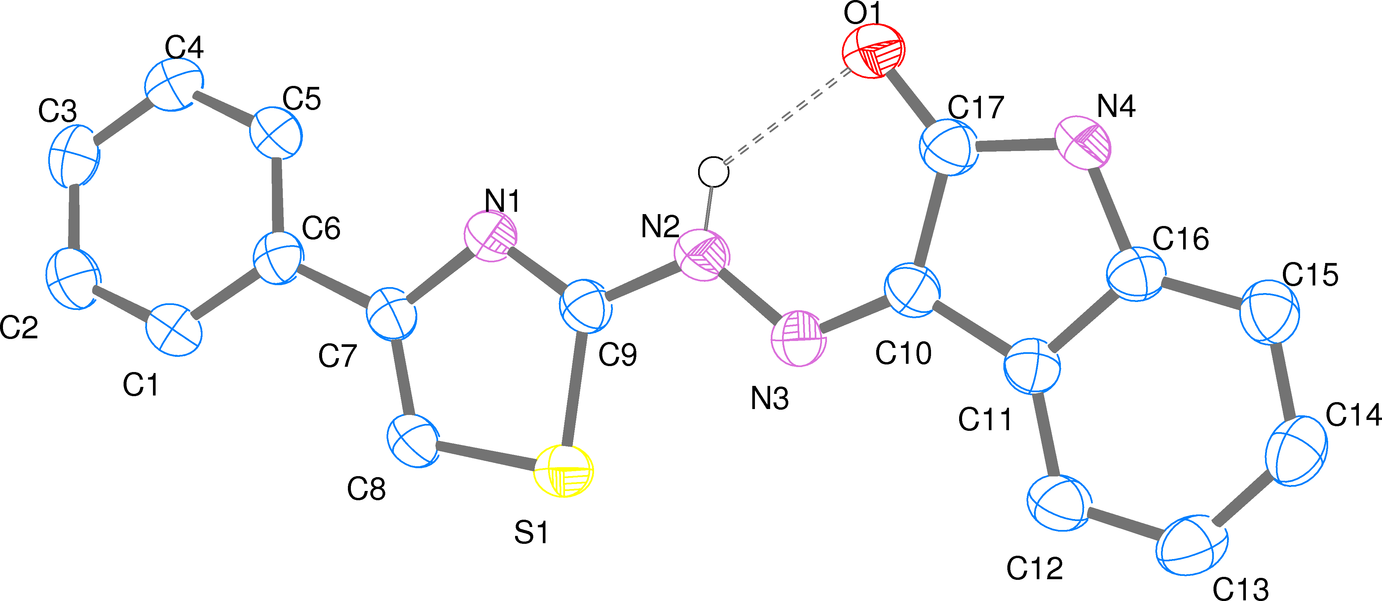

Supplement: Supplementary file 4 [file e-70-o1177-fig1.tif]

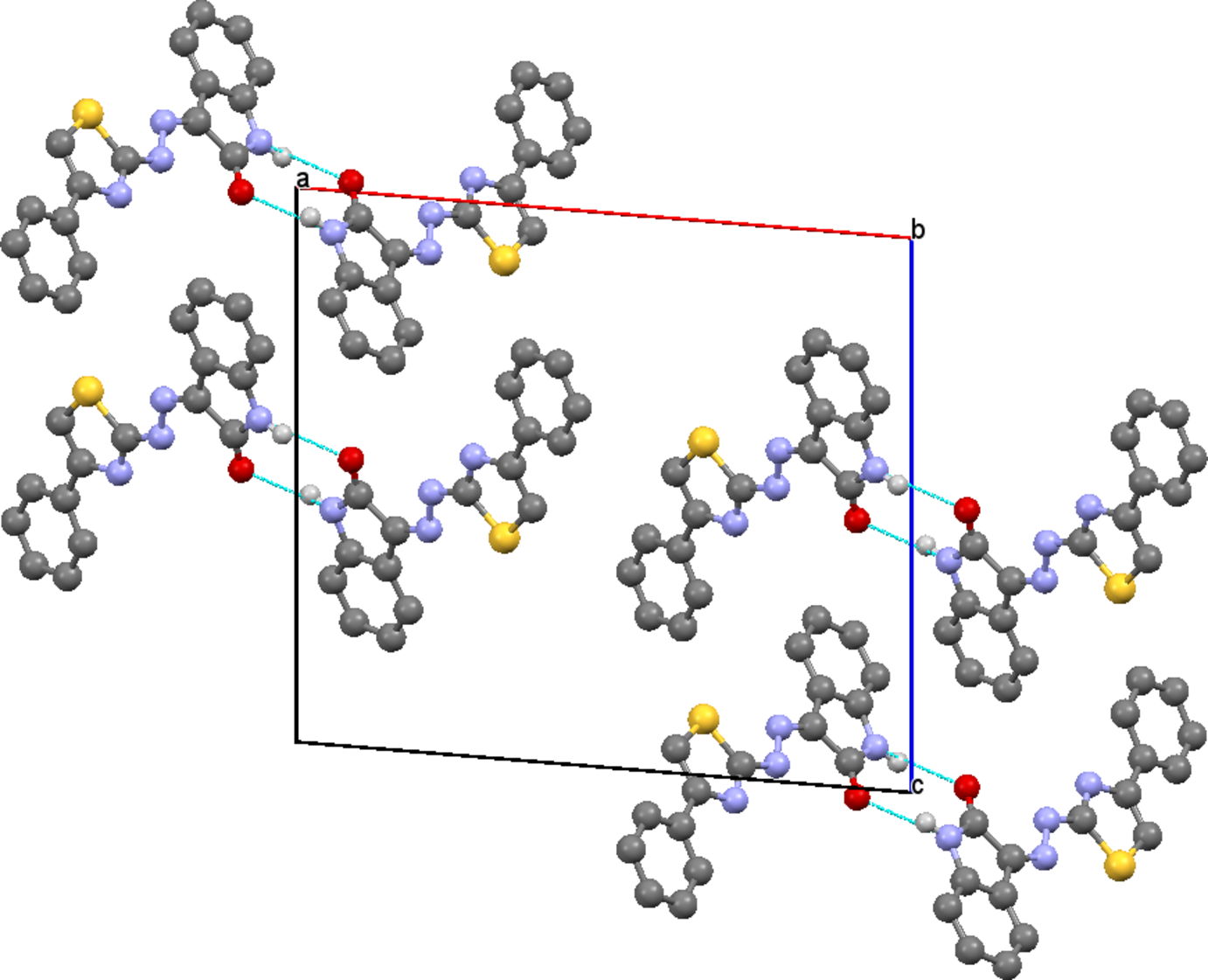

Supplement: Supplementary file 5 [file e-70-o1177-fig2.tif]
